# Correlation of P16 (Ink4a) and CK17 to HPV (16E6+18E6) in Premalignant and Malignant Lesions of Uterine Cervix: A Clinicopathologic Study

**Published:** 2016

**Authors:** Mohammed K. Chaloob, Alaa G. Hussein, Ban J. Qasim

**Affiliations:** 1 *Missan Health Directorate, Missan, Iraq *; 2 *Dept. of Pathology and Forensic medicine, College of Medicine, Al-Nahrain University, Baghdad, Iraq*

**Keywords:** LSIL, HSIL, cervical carcinoma, p16 (ink4a), CK17, HPV(16E6+18E6)

## Abstract

**Background:**

This research was accomplished to evaluate the IHC expression of p16 (ink4a) and CK17 in low grade cervical intraepithelial lesions (LSIL), high grade cervical intraepithelial lesions (HSIL) and invasive cervical carcinomas and to assess their correlation to HPV (16E6+18E6).

**Methods:**

The study included (127) formalin-fixed paraffin-embedded cervical biopsies; of which 22 cases were chronic cervicitis, 24 cases were LSIL, 28 cases were HSIL and 53 cases were invasive cervical carcinomas. Sections were immunohistochemically stained for p16 (ink4a), CK17 and HPV (16E6+18E6).

**Results:**

The study established a highly significant increase in IHC of expression of p16 (ink4a), CK17 and HPV (16E6+18E6) from LSIL through HSIL to invasive carcinomas (*P*-value˂0.001). There was non-significant association between IHC expression of all makers with age of patients; types, grade and stage of cervical carcinomas (*P*-value˃0.05). HPV (16E6+18E6) revealed a significantly positive correlation with p16 (ink4a) (*P*-value˂0.05) and a non- significant correlation with CK17 (*P*-value˃0.05); in LSIL, HSIL and invasive carcinoma cases.

**Conclusion:**

p16 (ink4a) expression directly reflects infection with high risk HPV in cervical lesions and can add a significant diagnostic accuracy in the evaluation of CIN. CK 17 is a good marker of malignant transformation, with increasing in its expression according to the severity of cervical lesions; however, it is not related to HPV infection. Both markers are not related to prognostic variables of patients with cervical carcinoma.

## Introduction

Cervical intraepithelial neoplasia (CIN) is a premalignant (dysplastic) lesion, which is marked with abnormal cell maturation and nuclear atypia (1). Majority of CIN lesions stay non-progressive, or are attacked by the host's defenses without therapy. Yet, a minority of lesions, without treatment, advance to invasive squamous cell carcinoma (SCC). The main etiological factor to CIN is chronic infection with high-risk human papilloma virus (HR-HPV) types 16 or 18 (2). Classification system has been lately customized to a simple two-tiered system, with low grade squamous intraepithelial lesion (LSIL) and high grade squamous intraepithelial lesion (HSIL) (3).

HR-HPV genotypes are essential for cervical cancer progression and its late precursor lesion, CIN-III. HPV type 16 is the most carcinogenic genotype, detected in about 60% of cervical cancers, followed by HPV18 found in nearly 15% of cervical cancers (4, 5).

 P16 (ink4a) is a cyclin dependent kinase inhibitor which coordinates transition from G1 to S phase of the cell cycle, acts as a tumor suppressor protein(6). P16 (ink4a) expression, which can be assessed immunohistochemically, is directly associated with HPV (7).Thus, this protein can help in the evaluation of CIN lesions (8).Cytokeratin 17 (CK17) is a type I (acidic) CK, its expression by mean of immunohistochemistry can assist in differentiating the grade of CIN lesions. In cervical carcinoma, CK 17 was always detected (9, 10).

The objective of this work was to study the expression of p16 (ink4a) and CK17 in LSIL, HSIL and invasive cervical carcinomas and to assess their correlation to HPV (16E6+18E6) expression in those lesions.

## Materials and Methods

This study enrolled 127 formalin fixed paraffin embedded cervical biopsies; of which 22 cases were chronic cervicitis, 24 cases were LSIL (CIN-I), 28 cases were HSIL (CIN-II, 21 and CIN-III, 7) and 53 cases were invasive cervical carcinomas retrieved from the archival materials of two teaching hospitals for the period from January 2012 to October 2014. 

This study was permitted by study Institutional Review Board of College of Medicine /Al-Nahrain University, which funded this study.

Twenty cases of LSIL were punch biopsies and 4 cases were cone biopsies. From cases of HSIL, 20 cases were punch biopsies, 6 cases were cone biopsies and 2 cases were total abdominal hysterectomy (TAH); for invasive cervical carcinomas 45 cases were TAH, 6 cases were punch biopsies and 2 cases were cone biopsy.

Clinicopathological data (age; grade of cervical neoplasia; histopathological type, grade and FIGO (International Federation of Gynecology and Obstetrics) pathological stage of cervical carcinomas) were collected from patients’ reports. 

From each paraffin block, 4 representative (4 micrometer) sections were obtained, one section was stained with Hematoxylin and Eosin (H&E) and revised by a pathologist and the remaining three were subjected to immunohistochemical testing for : anti-HPV (16 E6+18 E6) antibody, clone (C1P5), anti-P16 (ink4a) antibody, clone (2D9A12) and anti-CK 17 antibody, clone (E3); all manufactured by Abcam. 

**Interpretation of the results of immunohistochemical staining**

**HPV (16E6+18E6):**

Brown nuclear staining or combined nuclear and cytoplasmic staining is considered positive reaction. Cytoplasmic staining only was considered as negative (11, 12). Positive control is cervical carcinoma known to be positive for HPV (16 and/or 18). Scoring of IHC expression of HPV (16E6+18E6) was performed using an arbitrary semi-quantitative scale: (−), no staining observed representing negative staining; ˂25% representing mild positive staining (+); 25%–50% representing moderate positive staining (++) and >50% representing extensive (+++) immunostaining(13).

**P16 (ink4a):**

Brown nuclear and cytoplasmic staining is considered positive. Positive control is astrocytoma. Scoring was semi-quantitative based on the following:

 The intensity scales: 0=no staining; 1=weak focally positive; 2=strong, focally positive or weak, diffusely positive and 3= strong, diffusely positive. Percentage of positive nuclear staining scales: 1=1-25%, 2=26-50%, 3=51-75%, and 4=76-100%. Final score ranges from 0, 2-7 (14).

**CK17**

Brown cytoplasmic staining is considered positive. Positive control is squamous cell carcinoma. Technical negative control was obtained by omission of primary antibody. The IHC expression of CK 17 was assessed semi-quantitatively by taking the percentage of stained cells. Cases showing <5 % positive cells were regarded as negative, and those having > 5% positive cells were considered positive. Scores were counted as the following: mild (+) when 5–30 % positive cells, moderate (++) when 31–60 % positive cells and strong (+++) when more than 60 % positive cells (15).

Technical negative controls for all markers were prepared by exclusion of primary antibody.

**Statistical Analysis**

Statistics was accomplished with SPSS V. 17 (Chicago, IL, USA) and Excel 2007 programs. Continuous variables were calculated as mean ± SEM (standard error of the mean), while categorical variables were expressed as numbers and percentages. Statistical relations between two categorical variables were tested using Chi-square or Fisher exact tests. Relations between categorical and continuous variables were tested using unpaired t-test and ANOVA. The correlations between various markers were tested using Spearman rank correlation. 

## Results

The clinicopathological data of LSIL, HSIL and carcinoma cases are summarized in Table 1. 

**Table 1:** Clinicopathological data of LSIL, HSIL and invasive cervical carcinoma cases

**Clinicopathological data**		**Values**
Age :Mean(range + SEM)years	LSIL	38.46±2.3 (22-62)
	HSIL	43.32±1.92 (27-63)
	Invasive cervical carcinoma	44.13±1.4 29-70
Histopathological types of invasive cervical carcinoma	Adenocarcinomas	12 (22.64%)
	Adenosquamous carcinomas	3 (5.66%)
	Squamous cell carcinomas	38 (71.7%)
Grade of invasive cervical carcinoma	Well-differentiated	9 (16.98%)
	Moderately-differentiated	28 (52.83%)
	Poorly-differentiated	16 (30.19%)
Stage of invasive cervical carcinoma(pathological FIGO staging system)*	I	13 (29%)
	II	24 (53%)
	III	8 (18%)

SEM: standard error of the mean, LSIL: low grade squamous intraepithelial lesion, HSIL: high grade squamous intraepithelial lesion,

* 8 out of 53 carcinoma patients were lacking information about the stage (6 cases were punch biopsies and 2 cases were cone biopsies).

**HPV (16E6+18E6) immunohistochemical expression**

**Fig 1:**
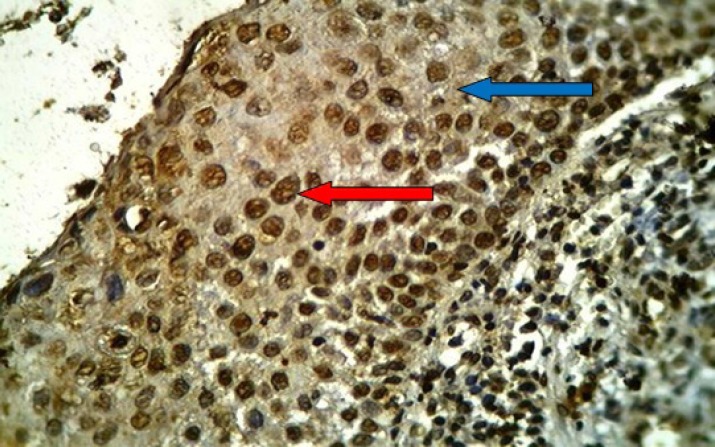
HSIL of uterine cervix stained immunohistochemically with anti-HPV (16E6+18E6) monoclonal antibody showing positive brown nuclear (red arrow) and cytoplasmic (blue arrow) staining with extensive immunostaining (+++), (40X

**Fig 2:**
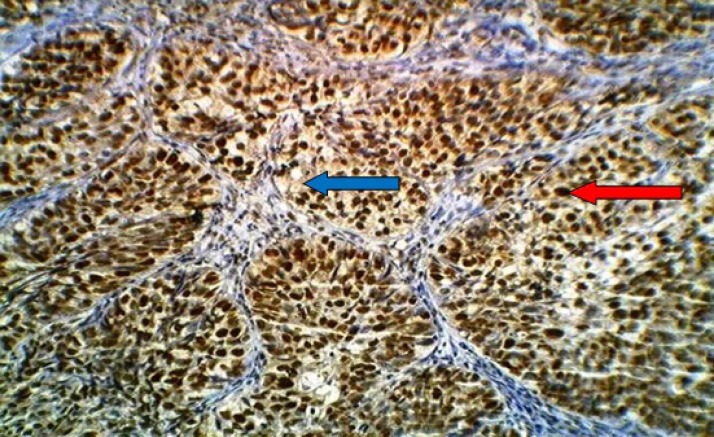
Moderately differentiated non-keratinizing squamous cell carcinoma of the uterine cervix stained immunohistochemically with anti-HPV (16E6+18E6) monoclonal antibodyshowing positive brown nuclear (red arrow) and cytoplasmic (blue arrow) staining with extensive immunostaining (+++), (20x

HPV (16E6+18E6) IHC was negative in all of the cases of chronic cervicitis, however, it was significantly increased in expression with rising severity of the lesions from LSIL to HSIL; and the highest expression was noted in carcinomas (*P*˂0.001). HPV (16E6+18E6) was expressed in 7 out of 24 LSIL cases (29.2%) while in HSIL, 13 cases out of 28 (46.4%) were positive for HPV (16E6+18E6) immunostaining (Figure 1). Positive HPV (16E6+18E6) expression was seen in 36 out of 53 carcinoma cases (67.9%) (Figure 2) .The difference in the distribution of cases according to scores of IHC expression of HPV (16E6+18E6) was highly statistically significant (*P*˂0.001) (Table 2).

**Table 2:** Frequency distribution of LSIL, HSIL and invasive cervical carcinoma cases according to immunohistochemical expression and scoring of immunostaining of HPV (16E6+18E6

**Frequency of HPV (16E6+18E6) expression and scores**	**LSIL** **No. (%)**	**HSIL** **No. (%)**	**Carcinoma** **No. (%)**
**Positive**	7 (29.2)	13 (46.4)	36 (67.9)
**Negative**	17(70.8)	15 (53.6)	17 (32.1)
**Mild(+)**	4 (16.7)	3 (10.7)	4 (7.5)
**Moderate(++)**	1 (4.2)	4 (14.3)	9 (17)
**Extensive(+++)**	2 (8.3)	6 (21.4)	23 (43.4)
**Total**	24	28	53
***P*** **-value**	˂0.001		

LSIL: low grade squamous intraepithelial lesion, HSIL: high grade squamous intraepithelial lesion, No.: number

Statistical analysis recorded non-significant association between age and IHC expression of HPV (16E6+18E6) in studied squamous intraepithelial lesion (SIL) and carcinoma cases, (*P*=0.558) and (*P*=0.518), respectively. There was also non-significant association of HPV (16E6+18E6) expression with histopathological types (*P*=1), grade (*P*=1) and FIGO pathological stage of invasive cervical carcinomas (*P*=0.442) (Table 3).

**Table 3:** Association of HPV (16E6+18E6) immunohistochemical expression with clinicopathological parameters of SILs, and invasive cervical carcinomas

Clinicopathological parameter		Positive HPV (16E6+18E6)	Negative HPV (16E6+18E6)	*P*- value
Age (yr) (mean±SEM)	SIL	39.85±2.55	38±1.9	0.558
	Invasive carcinoma	37.83±1.97	39.94±2.17	0.518
Histopathological type of invasive carcinomas	Adenocarcinoma No. (%)	8 (66.7%)	4 (33.3%)	1.000
	Adenosquamous No. (%)	2 (66.7%)	1 (33.3%)	
	Squamous No. (%)	26 (68.4%)	12 (31.6%)	
Grade of invasive carcinoma	Well-differentiated No. (%)	6 (66.7%)	3 (33.3%)	1.000
	Moderately-differentiated No. (%)	19 (67.9%)	9 (32.1%)	
	Poorly-differentiated No. (%)	11 (68.8%)	5 (31.2%)	
Pathological stage of invasive carcinoma	I No. (%)	8 (61.5%)	5 (38.5%)	0.686
	II No. (%)	15 (62.5%)	9 (37.5%)	
	III No. (%)	7 (87.5%)	1 (12.5%)	

SILs: squamous intraepithelial lesion, SEM: standard error of the mean,No.: number

**p16 (ink4a)immunohistochemical expression**

Descriptive analysis discovered a highly significant increase in the IHC expression of p16 (ink4a) from LSIL (37.5%) through HSIL (67.9%) to carcinomas (94.3%), (*P*˂0.001), (Table 4). None of the cases of chronic cervicitis showed positive immunoreactivity to p16 (ink4a).

**Fig 3:**
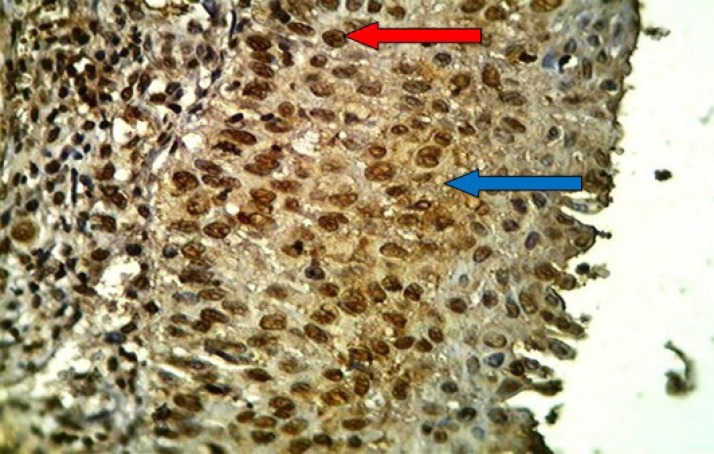
HSIL of the uterine cervix stained immunohistochemically with anti-p16 (ink4a) monoclonal antibody showing positive brown nuclear (red arrow) and cytoplasmic (blue arrow) staining with moderate intensity (2), high percentage (4) and score (6), (40X

**Fig 4:**
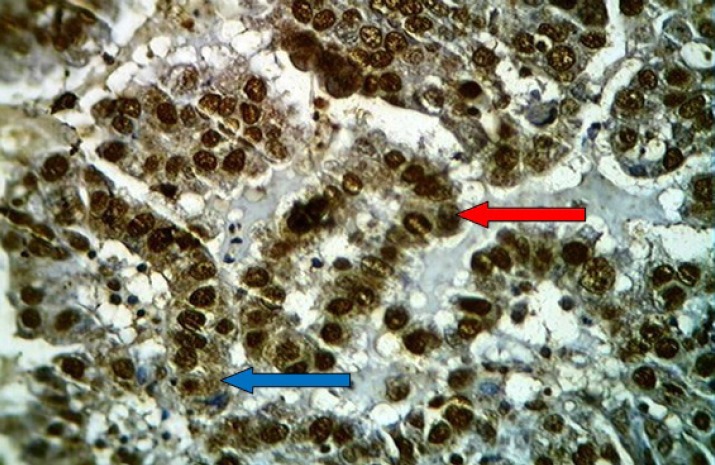
Moderately differentiated adenocarcinoma of the uterine cervix stained immunohistochemically with anti-p16 (ink4a) monoclonal antibody showing positive brown nuclear (red arrow) and cytoplasmic (blue arrow) staining with strong intensity (3) ,high percentage (4) and score (7), (40X

**Fig 5:**
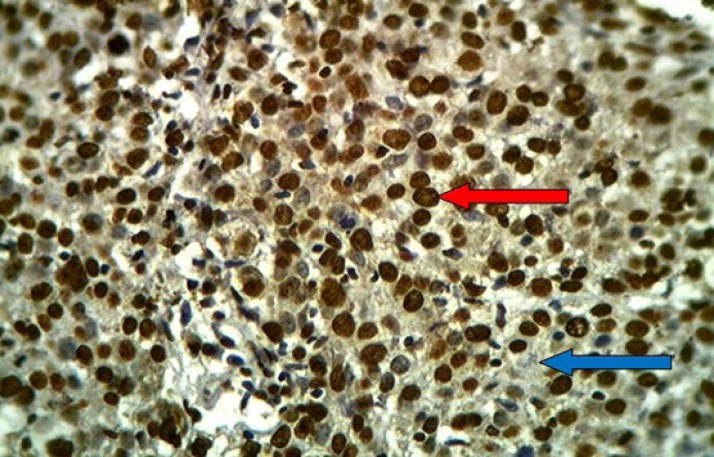
Poorly differentiated cell squamous carcinoma of the uterine cervix stained immunohistochemically with anti-p16 (ink4a) monoclonal antibody showing positive brown nuclear (red arrow) and cytoplasmic (blue arrow) staining with strong intensity (3) ,high percentage (4) and score (7), (40X

With reference to the score of p16 (ink4a) IHC expression; high scores(6-7) were noticed in 2 out of 24 LSIL cases (8.3%), 7 out of 28 HSIL cases (25%) (Figure 3) and 25 out of 53 cases of cervical carcinoma (47.2%) (Figures 4, 5). The difference in the frequency distribution of the cases is highly significant (*P*˂0.001) (Table 4).

**Table 4:** Frequency distribution of LSIL, HSIL and invasive cervical carcinoma cases according to immunohistochemical expression and scoring of immunostaining of p16 (ink4a

**Expression of p16(ink4a)** **and Scores**	**LSIL** **No. (%)**	**HSIL** **No. (%)**	**Carcinomas** **No. (%)**
**Positive**	9 (37.5)	19(67.9)	50 (94.3)
**Negative**	15 (62.5)	9 (32.1)	3 (5.7)
**2**	2 (8.3)	3 (10.7)	9 (17)
**3**	3 (12.5)	2 (7.1)	7 (13.2)
**4**	1 (4.2)	4 (14.3)	4 (7.5)
**5**	1 (4.2)	3 (10.7)	5 (9.4)
**6**	2 (8.3)	4 (14.3)	10 (18.9)
**7**	0 (0)	3 (10.7)	15 (28.3)
**Total**	24	28	53
***P*** **-value**	˂0.001		

LSIL: low grade squamous intraepithelial lesion, HSIL: high grade squamous intraepithelial lesion, No.: number

Results exposed non-significant association of p16 (ink4a) expression with age of patients in both SILs and invasive carcinomas, (*P*=0.378) and (*P*=0.247), respectively. p16 (ink4a) expressions were not statistically different according to histopathological types, grade and FIGO pathological stage of invasive cervical carcinomas, (*P*=1), (*P*=0.570), (*P*=0.107), respectively (Table 5).

**Table 5:** Association of p16 (ink4a) immunohistochemical expression with clinicopathological parameters of SILs, and invasive cervical carcinomas

Clinicopathological parameter		Positive p16 (ink4a)	Negative p16 (ink4a)	*P*- value
Age (years) (mean±SEM)	SIL	39.96±2.15	37.25±2.14	0.378
	Invasive carcinoma	38.08±1.56	45.67±4.06	0. 247
Histopathological type of invasive carcinomas	Adenocarcinoma No. (%)	11 (91.7%)	1 (8.3%)	1.000
	Adenosquamous No. (%)	3 (100%)	0 (0%)	
	Squamous No. (%)	36 (94.7%)	2 (5.3%)	
Grade of invasive carcinoma	Well-differentiated No. (%)	8 (88.9%)	1 (11.1%)	0.570
	Moderately-differentiated No. (%)	26 (92.9%)	2 (7.1%)	
	Poorly-differentiated No. (%)	16 (100%)	0 (0%)	
Pathological stage of invasive carcinoma	I No. (%)	11 (84.6%)	2 (15.4%)	0.107
	II No. (%)	24 (100%)	0 (0%)	
	III No. (%)	8 (100%)	0 (0%)	

SILs: squamous intraepithelial lesion, SEM: standard error of the mean,No.: number

**CK17 immunohistochemical expression**

**Fig 6:**
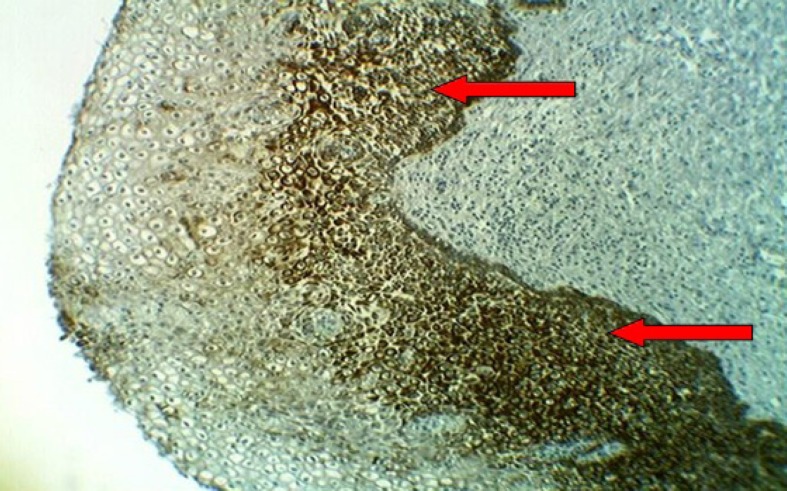
LSIL of the uterine cervix showing brown staining of cytoplasm with anti-Ck17 monoclonal antibody (arrows) with moderate intensity(++) into the lower third of the squamous epithelium, (10X

**Fig 7:**
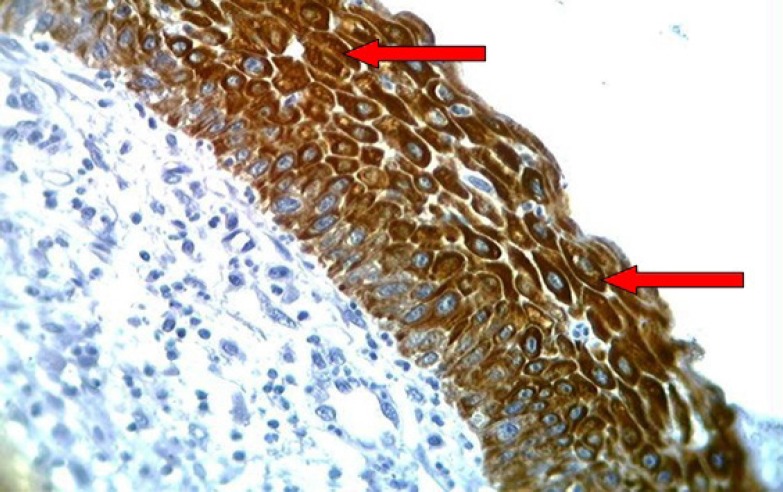
HSIL of the uterine cervix showing brown staining of cytoplasm with anti-Ck17 monoclonal antibody (arrows) with strong intensity (+++) of the whole thickness of the squamous epithelium, (40X

**Fig 8:**
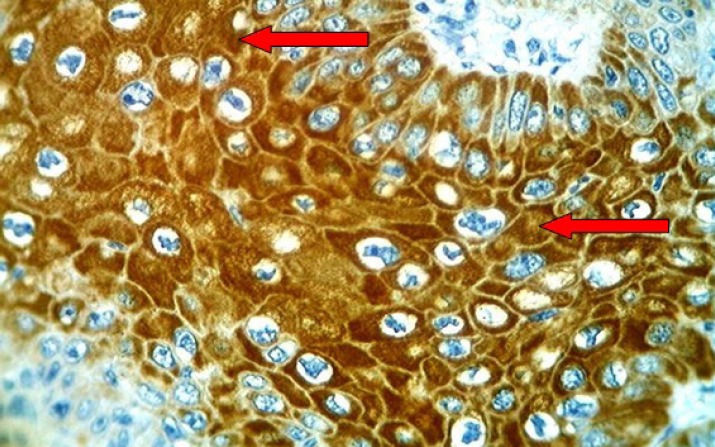
Cervical squamous cell carcinoma with moderate differentiation showing brown staining of cytoplasm with anti-Ck17 monoclonal antibody (arrows) with strong intensity (+++), (40X

CK17 showed a negative expression in all cases of chronic cervicitis with significant increment in its expression with rising severity of the lesions from LSIL (10 cases out of 24 (41.7%)) (Figure 6) through HSIL (20 cases out of 28 (71.4%)) (Figure 7) to carcinomas (48 cases out of 53 (90.6%)) (Figure 8) had been recognized; (*P*˂0.001), (Table 6).

**Table 6:** Frequency distribution of LSIL, HSIL and invasive cervical carcinoma cases according to immunohistochemical expression and scoring of immunostaining of CK17

**Expression of CK17 and** **scores**	**LSIL** **No. (%)**	**HSIL** **No. (%)**	**Carcinoma** **No. (%)**
**Positive**	10(41.7)	20(71.4)	48(90.6)
**Negative (0)**	14(58.3)	8 (28.6)	5 (9.4)
**Mild (+)**	8 (33.3)	2 (7.1)	7 (13.2)
**Moderate(++)**	2 (8.3)	11(39.3)	15 (28.3)
**Strong (+++)**	0 (0)	7 (25)	26 (49.1)
**Total**	24	28	53
***P*** **-value**	˂0.001		

LSIL: low grade squamous intraepithelial lesion, HSIL: high grade squamous intraepithelial lesion, No.: number

Concerning score of CK17 IHC staining, none of the cases of LSIL showed strong (+++) expression. In HSIL cases, CK17 reported strong expression in 7 cases (25%) (Figure 7) while CK17 was expressed strongly in 26 carcinoma cases (49.1%) (Figure 8). The difference is this distribution was highly statistically significant (*P*˂0.001) (Table 6).

Current research established a non- significant association between age and IHC expression of CK17 in studied SILs and carcinomas, (*P*=0.913) and (*P*=0.948), respectively. There was also non- significant difference in CK17 IHC expression among different histopathological types (*P*=0.691), different grades (*P*=0.442) and pathological FIGO stage (*P*=0.631) of cervical carcinoma (Table 7).

**Table 7:** Association of CK17immunohistochemical expression with clinicopathological parameters of SILs, and invasive cervical carcinomas

Clinicopathologicalparameter		Positive CK17	Negative CK17	P- value
Age (years) (mean±SEM)	SIL	38.57±1.8	38.91±2.68	0.913
	Invasive carcinoma	38.54±1.54	38.2±6.62	0.948
Histopathological type of invasive carcinomas	Adenocarcinoma No. (%)	10 (83.3%)	2 (16.7%)	0.691
	Adenosquamous No. (%)	3 (100%)	0 (0%)	
	Squamous No. (%)	35 (92.1%)	3 (7.9%)	
Grade of invasive carcinoma	Well-differentiated No. (%)	7 (77.8%)	2 (22.2%)	0.442
	Moderately-differentiated No. (%)	26 (92.9%)	2 (7.1%)	
	Poorly-differentiated No. (%)	15 (93.8%)	1 (6.2%)	
Pathological stage of invasive carcinoma	I No. (%)	11 (84.6%)	2 (15.4%)	0.107
	II No. (%)	22 (91.7%)	2 (8.3%)	
	III No. (%)	8 (100%)	0 (0%)	

SILs: squamous intraepithelial lesion, SEM: standard error of the mean,No.: number

**Correlation between of HPV (16E6+18E6) and p16 (ink4a), CK17 IHC expressions in SIL and carcinoma cases**

 In SIL cases, HPV (16E6+18E6) expression significantly correlated with the expression of p16 (ink4a), (*P*˂0.001). Nevertheless, CK17 showed non-significant with of HPV (16E6+18E6), (*P*=0.067) (table 8).

Pertaining to cervical carcinoma cases, results distinguished a significant correlation between IHC expression of HPV (16E6+18E6) and IHC expression of p16 (ink4a) (*P*˂0.001). The correlation between HPV (16E6+18E6) and CK17 IHC expression was non-significant (*P*=0.775) (Table 8).

**Table 8:** Correlation between HPV (16E6+18E6) and p16 (ink4a), CK17 IHC expressions in SIL and cervical carcinoma

**Other markers**	**HPV (16E6+18E6) in SIL**		**HPV (16E6+18E6) in carcinoma**	
	**r**	***P***	**r**	***P***
**p16(ink4a)**	0.869	˂0.001	0.939	˂0.001
**CK17**	-0.256	0.067	0.040	0.775

SILs: squamous intraepithelial lesion

## Discussion

In Iraq, during the period 1976-1985, the cervical cancer ranked the sixth among the commonest 10 cancers in females (16), whereas throughout the period 1995-1997, it ranked the tenth within the leading cancer in females. Cervical cancer constitutes 1.4% of the total number of cancers with annual number of 113 new cases reported in 1995, 1996, and 1997, respectively (17). Nowadays and according to the latest Iraqi cancer registry, 2011, cervical cancer is out of the commonest ten cancers in Iraqi females (18).

Although this study is not a large epidemiological one that expresses the prevalence and incidence of HPV (16+18) according to different clinicopathological parameters of cervical lesions; but in general HPV (16E6+18E6) was significantly increased in its expression with mounting severity of the lesions from LSIL (29.2%) through HSIL (46.4%) to carcinomas (67.9%). 

 Activity of HPV infection is determined through early gene products’ expression to be active or latent or leads to malignant transformation (19).The E6 protein targets the degradation of p53 and thereby conquering the cellular arrest and p53 proapoptotic activities (20). One of the mechanisms of p53 inactivation is through the inhibition of binding of p53 to its target gene sequence (21). 

In an Iraqi study completed in Baghdad using ISH technique (22), HPV (16) was recovered in 40% of SIL cases without detection of HPV (18) and in 75% of carcinoma cases. In another Iraqi study using PCR technique (23), HPV (16+18) was detected in SILs and squamous cell carcinomas in 50% and 60%, respectively.

In an Iranian meta-analysis study (including 20 studies) (24), the prevalence of HPV (16+18) in Iranian female patients with normal cervix, LSIL, HSIL and invasive carcinomas were (4.9%), (53.4%), (64.5%) and (64.5%), respectively. In Saudi Arabia using PCR technique, the prevalence of HPV (16+18) in invasive cervical cancer was (78.7%) and (74.5%), respectively (25, 26). In a Turkish research, HPV-16 and HPV-18 were detected in 41.4% and 10.5% of LSIL, respectively; and in 68.8% and 4.2% of HSIL, correspondingly (27).

In an Indian works (28), and Sowjanya et al. (29), using PCR technique, the HPV (16+18) were detected in 79.3% and 86.1% of cervical carcinomas, respectively. In a Brazilian research using PCR technique, HPV-16 and HPV-18 were detected in 77.6% and 12.3% of cervical carcinomas, respectively (30).

In a retrospective international survey including 38 countries during the period 1949-2009, the prevalence of HPV (16+18) in invasive cervical cancer detected by PCR technique in Europe, North America (USA), central South America, Africa, Asia and Australia were 73%, 79%, 68%, 71%, 71% and 79%, in that order (7).

The result of the current article is almost parallel to other Iraqi studies and studies done in neighboring Islamic countries. The frequency of HPV (16+18) in the current study is lower than other countries like Brazil, India, Turkey, Europe, USA and Australia and this is possibly due to difference in sample size, use of more sensitive methods for detecting HPV (16+18) like ISH and PCR compared to IHC method which was used in this study and may be attributable to the unique religious and cultural restrictions preventing women to have multiple sexual partners in Iraq, thus reducing unsafe sexual relationships therefore lowering the threat of HPV infection.

This study reported non-significant connection of age and HPV (16E6+18E6) expression in studied SIL and carcinoma cases. This outcome is regular with other authors (29, 31). The prevalence of HR-HPV was more common in younger age women, this difference could be attributed to environmental, racial and different age at first intercourse (30).

The early age of the first sexual practice is unquestionably related to HPV infections (32). The immature transitional zone of the developing cervix (perimenarche) or cervix healing from delivery, trauma or infections, are at high risk for an HPV infection (33).

The current research showed IHC expression of HPV (16E6+18E6) in 66.7% of adenocarcinomas, 66.7% of adenosquamous carcinomas and in 68.4% of squamous carcinomas with no significant difference (P=1). Those findings are in accordance to another Iraqi article (26, 30).

There was non-significant difference in IHC expression of HPV (16E6+18E6) between the grade and pathological stage of studied cervical carcinoma cases in the present study. This result is accordance with the results obtained from other studies (34-36).

P16 (ink4a) was also significantly increased in its expression from LSIL (37.5%) through HSIL (67.9%) to carcinomas (94.3%). This result is in agreement with several other studies (37-42). In a study done on 59 cases of different cervical lesions(14), no significant difference in IHC expression of p16 (ink4a) between CIN lesions and cervical carcinomas had been observed, this discrepancy is probably due to ecological, cultural and geological variations, as well as sample size. 

p16 overexpression could reveal the potential for cervical malignant transformation as it is augmented with higher CIN grade (38). In cervical lesions induced by HPV, viral oncoprotein E7 interacts and inactivates pRb. Consequently, inactivated pRb passes the cell cycle checkpoint G1/S with no difficulty. Functionally active gene Rb is found to be a factor in the negative regulation of the expression of INK4a on a transcriptional level (43,44). Also rising p16 expression may well reflect additional strong inactivation of pRb by HR-HPV that commonly result in progression of CIN (45).

Concerning the age, the current work revealed no significant association between age and IHC expression of p16 (ink4a) in both studied SILs and carcinoma cases. This data is in harmony with other authors (14).

On the subject of cervical carcinoma cases, the present study recorded non- significant difference in IHC expression of p16 (ink4a) among different histopathological types, grade and pathological stage of studied cervical carcinoma cases. These results were also observed by other researches (14, 40).

The current work established that p16 (ink4a) is highly correlated with HPV (16E6+18E6) in SILs and cervical carcinoma cases, respectively. This result is akin to that obtained by other studies (46-49).

P16 (ink4a) is a cell cycle inhibitor that binds to cdk4 and cdk6 that inhibits pRb phosphorylation and inactivation (50).The down regulation of p16 (ink4a) is linked to the pathogenesis of cancers in various organs (51, 52).Yet, overexpression of p16 (ink4a) is usually found in premalignant cervical lesions and cancers (53).

Through a negative feedback mechanism, E6 and E7 proteins can cause induce production of p16 (ink4a), as they bind to pRb and inactivate it (53). HPV-E7 proteins leads to functional inactivation of pRb and thereby causing p16 (ink4a) expression in cervical lesions (50). Using a panel of p16 (ink4a)- specific monoclonal antibodies, p16 (ink4a) is specifically overexpressed in CIN lesions infected with HR-HPV and in carcinomas, but not in normal cervix or inflammatory lesions (54).

CK 17 is present in cervical reserve cells (from which CIN is originated). Its expression in term of percentage and intensity is increase with increasing severity of CIN. In cervical carcinoma, keratin 17 was always detected, so the development of carcinoma from CIN-III is restricted to CK17 positive CIN-III (9, 10).

This work set up positive expression of CK 17 in all cases of SIL and carcinomas, with significant augment in its expression with increasing severity of the lesions from LSIL (41.7%) through HSIL (71.4%) to cervical carcinomas (90.6%). This result is nearly analogous to that obtained earlier (55),who found IHC expression of CK17 in 33.3% of CIN-I, 58.1% of CIN-II, 81.4% of CIN-III and in 95.2% of squamous cell carcinoma. In a research (56), CK17 was not found in non-neoplastic cervix and its expression was increased significantly from CIN-III to invasive carcinoma. CK 17 was detected in the lowermost parts of CIN lesions with different grades in up to half of cases with its higher expressions in reserve cells and reserve cell hyperplasia where cervical epithelial stem cells reside assessing the importance of CK 17 as stem cell marker (57).

Taking age into consideration, this study reported non- significant association between age and IHC expression of CK17 in SILs and carcinomas, that is in tune with Ikeda et al., 2008 (55).

The current paper showed non- significant difference in IHC expression of CK17 among different histopathological types, grade and pathological stage of cervical carcinoma that is in unity with other authors; (Carrilho et al., 2004) (56), and (Maddox et al., 1999) (58).

CK 17 was not correlated to IHC expression of HPV (16E6+18E6) in the studied SILs and carcinoma cases, that is in concord with other studies (46, 59). Missing the correlation of CK 17 with HPV (16+18) indicates that this marker is of cervical dysplastic tissues and not of cervical infection by HPV (59).

## Conclusion

p16 (ink4a) expression directly reflects infection with high risk HPV in cervical lesions and can add a significant diagnostic accuracy in the evaluation of CIN. CK 17 is a good marker of malignant transformation, with increasing in its expression according to the severity of cervical lesions; however, it is not related to HPV infection. Both markers are not related to prognostic variables of patients with cervical carcinoma. 
